# Mapping optogenetically-driven single-vessel fMRI with concurrent neuronal calcium recordings in the rat hippocampus

**DOI:** 10.1038/s41467-019-12850-x

**Published:** 2019-11-20

**Authors:** Xuming Chen, Filip Sobczak, Yi Chen, Yuanyuan Jiang, Chunqi Qian, Zuneng Lu, Cenk Ayata, Nikos K. Logothetis, Xin Yu

**Affiliations:** 1 Research Group of Translational Neuroimaging and Neural Control, High-Field Magnetic Resonance, Max Planck Institute for Biological Cybernetics, 72076 Tuebingen, Germany; 2 University of Tuebingen, 72074 Tuebingen, Germany; 3 Department of Neurology, Wuhan University, Renmin Hospital, Wuhan, 430060 China; 4 Graduate Training Centre of Neuroscience, International Max Planck Research School, University of Tuebingen, 72074 Tuebingen, Germany; 5 Athinoula A. Martinos Center for Biomedical Imaging, Massachusetts General Hospital and Harvard Medical School, Charlestown, 02129 MA USA; 6 Department of Radiology, Michigan State University, East Lansing, 48824 MI USA; 7 Neurovascular Research Laboratory, Department of Radiology, Massachusetts General Hospital, Harvard Medical School, Charlestown, 02129 MA USA; 8 Stroke Service and Neuroscience Intensive Care Unit, Department of Neurology, Massachusetts General Hospital, Harvard Medical School, 02129, Boston, USA; 9 Department of Physiology of Cognitive Processes, Max Planck Institute for Biological Cybernetics, Tuebingen, 72076 Germany; 10 Department of Imaging Science and Biomedical Engineering, University of Manchester, Manchester, M13 9PT UK

**Keywords:** Imaging, Optogenetics, Neuro-vascular interactions

## Abstract

Extensive in vivo imaging studies investigate the hippocampal neural network function, mainly focusing on the dorsal CA1 region given its optical accessibility. Multi-modality fMRI with simultaneous hippocampal electrophysiological recording reveal broad cortical correlation patterns, but the detailed spatial hippocampal functional map remains lacking given the limited fMRI resolution. In particular, hemodynamic responses linked to specific neural activity are unclear at the single-vessel level across hippocampal vasculature, which hinders the deciphering of the hippocampal malfunction in animal models and the translation to critical neurovascular coupling (NVC) patterns for human fMRI. We simultaneously acquired optogenetically-driven neuronal Ca^2+^ signals with single-vessel blood-oxygen-level-dependent (BOLD) and cerebral-blood-volume (CBV)-fMRI from individual venules and arterioles. Distinct spatiotemporal patterns of hippocampal hemodynamic responses were correlated to optogenetically evoked and spreading depression-like calcium events. The calcium event-related single-vessel hemodynamic modeling revealed significantly reduced NVC efficiency upon spreading depression-like (SDL) events, providing a direct measure of the NVC function at various hippocampal states.

## Abstract (web summary)

Detailed characterization of large-scale hemodynamic responses linked to specific neural activity remains to be elucidated at the single-vessel level across the subcentimeter scale hippocampal vasculature in vivo. Here, authors use a novel multi-modal fMRI platform to characterize distinct spatiotemporal patterns of hippocampal hemodynamic responses that were correlated to the optogenetically evoked Ca2+ events and to further demonstrate the significantly reduced neurovascular coupling efficiency upon spreading depression-like Ca^2+^ events.

## Introduction

Over the past few decades the combination of behavioral and psychophysical studies with anatomical, pharmacological, and functional magnetic resonance imaging (fMRI), permitting whole-brain mapping of brain networks, has expanded our understanding of brain function and occasionally dysfunction. The blood oxygen level-dependent (BOLD) fMRI^1–4^, in particular, is now used as a standard tool for demarcating brain states, and potentially, dynamic transitions from one state to another^5–9^. Nonetheless, fathoming into the true and eventually detailed neural mechanisms underlying the BOLD positive and negative responses, more so at the level of cortical microcircuits and deep brain nuclei, is currently still extremely difficult if not impossible. Conventional fMRI yields surrogate signals such as continuous blood flow, volume, and oxygenation changes^1–4,10,11^. These indirect functional mapping schemes cannot differentiate between function-specific processing and neuromodulation, between bottom-up and top-down signals, occasionally confusing even excitation and inhibition, depending on the circuit-dependent direct or indirect nature of local neural activity^5,12–15^. The origin of such problems is not only due to the weak spatial specificity of the fMRI signal to its neural source but also to the very fact that the exact relationship between the metabolic/hemodynamic responses and the underlying neural activity patterns remains mostly elusive. Using high-resolution fMRI methods to map the animal brain, BOLD and cerebral blood volume (CBV) fMRI signals can be detected from individual venule and arteriole voxels from deep cortical layers^16–18^. Beyond the in vivo penetrating depth of conventional optical imaging, single-vessel fMRI methods have enabled direct measurement of vessel-specific hemodynamic responses with fMRI in a large spatial scale to interpret better the neurovascular coupling (NVC) contribution to the fMRI signal acquired in deep brain regions.

Simultaneous fMRI and electrophysiological recordings offered the first insights into the NVC underlying the cortical fMRI signal in both task-related and resting-state conditions^19,20^. Lately, genetically encoded Ca^2+^ indicators, for example, GCaMP, mediating Ca^2+^ imaging from neurons or astrocytes have also been combined with optical hemodynamic imaging or fMRI, demonstrating various NVC patterns across multiple scales at different cortical states^21–28^. Besides the multi-modal correlation analysis of cortical dynamic signals, the highly varied global correlation of the fMRI signal to the concurrent hippocampal ripple activity has also demonstrated region-specific cortical NVC patterns^29^. In contrast to extensive cortical NVC studies to interpret the fMRI signal acquired in the cortex, the linkage between subcortical NVC events to the fMRI signal, for example, in the hippocampus, has not been well elucidated. Not only has the 3D location of the hippocampus in the brain restricted its accessibility to conventional optical imaging methods but also the mesoscale hippocampal vasculature has been seldom specified for hemodynamic mapping with fMRI. Previous in vivo hippocampal functional imaging studies applied micro-lens/micro-prism through the cortical tissue or removed the cortex above the hippocampus^30–33^. The optical fiber has been used to target the hippocampus for the measurement of Ca^2+^ from individual cell types or for fast dynamic recordings^34–38^. Using long-wavelength light pulses for deeper tissue penetration, three-photon microscopy has further expanded the optical penetration depth for NVC imaging of dorsal hippocampal CA1 regions in mice with a much less invasive surgical procedure in the mouse brain^39–41^. Nevertheless, it remains challenging to detect subcortical NVC events in animals with larger brains, such as rats and non-human primates, using multi-photon microscopic imaging methods. Although rodent hippocampal vasculature has been well described in histological studies by Coyle^42,43^ in the mid 1970s, no multi-modality neuroimaging studies have been performed to decipher the detailed vessel-specific hemodynamic responses throughout the hippocampal vasculature with fMRI and concurrent neuronal activity measurement in the hippocampus.

Here, we developed a multi-modal fMRI platform, aiming to specify the properties of NVC across the rat hippocampus. The experiments were performed in a high magnetic field scanner (14.1 T), with customized radiofrequency (RF) coils, and the balanced steady-state free procession (bSSFP) method that permits the acquisition of the fMRI signal from individual cortical penetrating arterioles and venules^22^, thereby expanding the line-scanning-based method for real-time single-vessel fMRI mapping^18,44,45^. This high spatial resolution vessel-specific fMRI mapping method allowed to directly measure mesoscale hemodynamic responses of the hippocampal vasculature. In particular, we applied the single-vessel fMRI to map the BOLD and CBV-weighted fMRI signal from interleaved arterioles and venules in the rat hippocampus, of which detailed vascular hemodynamic responses were imaged with a high spatiotemporal resolution. This has not been accomplished by other existing non-invasive global functional neuroimaging methods. This work provides direct evidence to show the deep brain large-scale hemodynamic vascular mapping with single-vessel fMRI beyond the penetration depth of conventional optical imaging methods. Using implanted optical fibers, optogenetically evoked neuronal Ca^2+^ and the spreading depression-like Ca^2+^ (SDL-Ca^2+^) events were detected with simultaneous single-vessel BOLD fMRI, demonstrating distinct spatiotemporal features of vascular hemodynamic responses. The varying NVC efficiency (NVCe) can be estimated by directly modeling single-vessel fMRI responses to concurrent Ca^2+^ events across the hippocampal vasculature. The simultaneous single-vessel hippocampal fMRI and Ca^2+^ recording not only provides a multi-modal platform for specifying the multi-scale NVC in the hippocampus but also sheds light on future pathological hippocampal NVC studies in disease animal models with stroke, epilepsy, and Alzheimer’s disease.

## Results

### Multi-modal hippocampal fMRI and local field potential

**Fig. 1:**
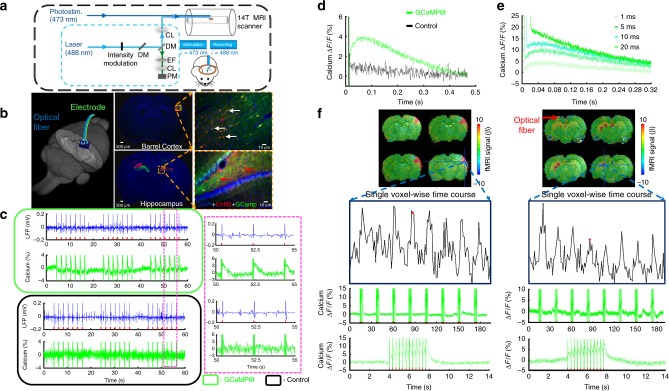
Optogenetically evoked Ca^2+^ recording with LFP or fMRI. **a** Schematic drawing for the light path of optogenetic activation and calcium recordings in the multi-modal fMRI platform (PM, photomultiplier; EF, emission filter; DM, dichroic mirror; CL, coupling lens). **b** Schematic drawing of optical fibers implantation to target the rat hippocampus in a 3D view (left). Channelrhodopsin (ChR2, red) and GcaMP6f (green) were co-expressed in the barrel cortex (BC, upper, white arrows) and hippocampus (lower, red arrows) with enlarged images (dashed box, right). **c** Simultaneous LFP (blue) and neuronal Ca^2+^ (green) traces in the hippocampus following optical stimulation (10 ms light pulse, 1 Hz, 7 s, 4 mW; upper: GCaMP6f expression; lower: control; right panel, enlarged view). **d** Averaged traces of optogenetically evoked Ca^2+^ spikes in the hippocampus (green: GCaMP6f expression; black: control). **e** Averaged traces of optogenetically evoked Ca^2+^ spikes with different widths of the light pulse (1, 5, 10, and 20 ms). **f** A representative color-coded BOLD fMRI map from the BC (left) and hippocampus (optical fiber insertion trace: red arrow), together with associated fMRI time courses (lower, top) and concurrent neuronal Ca^2+^ signals (lower, middle) in the block-design paradigm (illumination: 10 ms light pulse, 3 Hz, 4 s, 5 mW, zoomed views are averaged evoked Ca^2+^ signals from one epoch)

To study the hippocampal NVC with the multi-modal fMRI platform (Fig.1a), we co-expressed channelrhodopsin-2 (ChR2) and the genetically encoded Ca^2+^ sensor, GCaMP6f, in the rat hippocampus using adeno-associated viral (AAV) vectors (AAV5.Syn.GCaMP6f.WPRE.SV40; AAV5.CAG.hChR2-mCherry.WPRE.SV40). Figure 1b shows neurons labeled with either ChR2-mcherry or GCaMP6f in both barrel cortex (BC) and hippocampus for optogenetic fMRI with concurrent Ca^2+^ signal recording. First, optogenetically evoked local field potential (LFP) and GCaMP-mediated Ca^2+^ signals were simultaneously detected in the hippocampus of rats at varied light pulse widths, power levels, and frequencies (Fig. 1c, d, Supplementary Fig. 1). It is noteworthy that the optical light pulse introduced large artifacts for the GCaMP-mediated Ca^2+^ fluorescent signal detection. Given the specific temporal feature of optogenetically evoked Ca^2+^ transients, artifacts detected by the fast-sampling silicon photomultiplier (SiPM) can be distinguished easily from Ca^2+^ transients given its short light pulse duration. Figure 1d, e show the peak fluorescent Ca^2+^ signal at ~50–60 ms after the onset of the optogenetic light pulses with various widths from 1 to 20 ms, which is consistent with previous observations in the cortex^21^. This result demonstrates the feasibility of hippocampal optical fiber Ca^2+^ recordings with optogenetic stimulation.

Next, we aimed to verify the multi-modal fMRI platform in combination with both optogenetic stimulation and simultaneous Ca^2+^ recordings. Figure 1f demonstrates the optogenetically activated BOLD fMRI signals at the BC and the hippocampus with the 3D echo planar imaging fMRI (EPI-fMRI) method^46,47^. Both evoked BOLD fMRI signals from activated brain voxels and concurrent Ca^2+^ transients from nearby neurons can be detected using the block-design optogenetic stimulation paradigm. These multi-modal NVC events were acquired across spatial scales from the sub-millimeter scale neuronal ensembles surrounding the fiber tip to the macroscopic vascular hemodynamic response detected by fMRI. It is important to note that direct light pulse exposure on the naive rat hippocampus did not cause detectable positive BOLD fMRI signal through hippocampal vasculature due to the local blood flow regulation, that is, cerebral blood flow changes, as previously reported by ultrasound Doppler signal measurement^48^. The high power light pulse (>25 mW from 200 µm fiber tip) caused the focal negative fMRI signal near the fiber tip due to heating-induced susceptibility changes (frequency offset) (Supplementary Fig. 2)^49^. In contrast, the optogenetic stimulation of rats with ChR2 expression in the hippocampus evoked the strongest signals at the choroid plexus located at the dorsal wall of the lateral ventricle across multiple slices, containing draining veins from the hippocampus in a sub-centimeter scale away from the optical fiber tip (Fig. 1f). Although these results indicate that the direct effect of light exposure on the flow regulation contributes less to the measurable BOLD signal, the widely spread hemodynamic responses in hippocampal vasculature upon optogenetic stimulation remain poorly characterized with EPI-fMRI given the limited spatial resolution. These results also led to implementing the high-resolution single-vessel fMRI method into the multi-modal fMRI platform for hippocampal NVC mapping.

### Optogenetic hippocampal fMRI with ChR2 variant (C1V1)

It should be noted that in order to reduce the spectral wavelength overlap of GCaMP-based fluorescent signal excitation and optogenetic light pulse stimulation at 473 nm, we additionally applied the ChR2 variant (C1V1) to switch the optogenetic light pulse to 590 nm. Supplementary Figure 3 demonstrates similar dynamic patterns of the concurrent LFP or fMRI signal and fiber optic Ca^2+^ signal upon C1V1-mediated optogenetic activation in the rat hippocampus. These results further verify the feasibility of detecting optogenetically driven BOLD and intracellular neuronal Ca^2+^ signals in the hippocampus with the multi-modal fMRI platform.

### Optogenetic single-vessel hippocampal BOLD and CBV fMRI

**Fig. 2:**
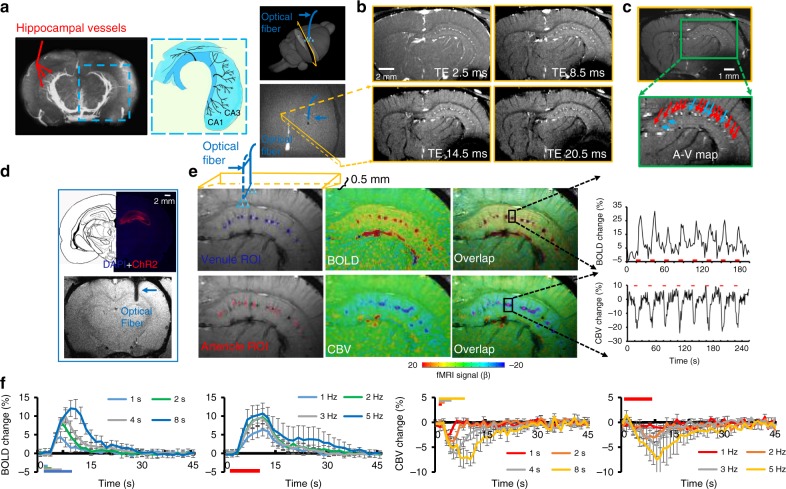
Single-vessel hippocampal BOLD and CBV-weighted fMRI. **a** The magnetic resonance angiography (MRA) image shows major vascular branches penetrating the rat hippocampus (middle image is the schematic drawing of the hippocampal transverse plane of vessels aligned in parallel [modified from Peter Coyle (1976)]). The 3D view of the 2D slice alignment to cover the transverse hippocampal structure (lower image is the horizontal view to show the 2D slice with 40° to the midline to cross the penetrating vessels, dark hole: the fiber optic tip). **b** Representative images of 2D MGE slices from the hippocampus at different TEs. **c** A–V map derived from images in **b**. Arterioles and venules appear as bright and dark dots, respectively [zoomed view of hippocampal arterioles (bright dots, red arrows) and venules (dark dots, blue arrows)]. **d** A histological section shows ChR2 expressed in the hippocampus (upper). The T2*-weighted (T2*-W) image shows the optical fiber inserted into the hippocampus (lower, blue arrow). **e** Venule (blue)/arteriole (red) ROIs on A–V maps (left). Evoked BOLD (upper) and CBV-weighted (lower) fMRI maps on the same 2D slice (center) and overlap (active voxels are in purple in overlap images). Time courses of the evoked BOLD and CBV-weighted fMRI with the block-design paradigm from a representative venule (upper) and arteriole (lower) ROI (illumination: 10 ms light pulse, 3 Hz, 4 s, 5 mW). **f** Averaged BOLD (upper) and CBV-weighted (lower) fMRI responses from different stimulation durations (1, 2, 4, and 8 s) and frequencies (1, 2, 3, and 5 Hz) (*n* = 4, mean ± SEM)

As shown with the magnetic resonance angiography (MRA) imaging in Fig. 2a, the hippocampal vasculature is aligned in parallel branches supplying blood to the saddle-shaped structure of the hippocampus^50^ (Fig. 2a, Supplementary Movie 1). To visualize individual vessels, we applied a 2D multiple gradient echo (MGE) slice transecting the parallel hippocampal vascular branches with 40° angle to the midline (Fig. 2a). Similar to previous single-vessel MRI studies in the cortex^18,22^, the 2D MGE images were acquired at different time of echo (TE) to distinguish individual arteriole and venule voxels from the surrounding parenchyma voxels enriched with capillaries. At shorter TE, due to in-flow effects from vessels at the short time of repetition (TR), all vessel voxels appear brighter than the surrounding voxels based on the T1-weighted MR contrast; however, at the longer TE, the fast T2* decay of the deoxygenated blood leads to darker signal intensity in venule voxels only (Fig. 2b, Supplementary Movie 2)^18^. Thus, by integrating the MGE images acquired at different TEs, we could distinguish individual hippocampal arterioles (bright dots) and venules (dark dots) from the anatomical single-vessel 2D map (arteriole–venule (A–V) map), showing the interleaved arterioles and venules in the hippocampus (Fig. 2c).

One essential improvement of this work is to apply the single-vessel bSSFP-fMRI to detect the optogenetically evoked fMRI signal in the rat hippocampus, demonstrating the deep brain single-vessel hemodynamic mapping with fMRI beyond the penetration depth of conventional optical imaging methods. Following the BOLD fMRI experiment, the CBV-weighted single-vessel fMRI was performed after the intravenous MION (iron oxide particle) injection. Figure 2d shows the optical fiber targeting the hippocampal CA1 region expressing ChR2. Upon optogenetic activation, peak BOLD signals were primarily overlapping with venule voxels, showing positive BOLD signals from individual venules. Peak CBV-weighted signals were located at arteriole voxels, showing negative CBV-weighted signals from individual arterioles in the hippocampus (Fig. 2e, Supplementary Movie 3). Besides the CBV-weighted single-vessel fMRI maps, we also calculated the CBV percentage change (%) map based on BOLD fMRI time courses acquired before the injection of MION particles^51^, showing positive %CBV changes from individual arterioles (Supplementary Fig. 4). Figure 2f shows vessel-specific mean hemodynamic BOLD and CBV-weighted responses upon the optogenetic stimulation at varying durations and frequencies of light pulses, demonstrating highly robust optogenetically driven single-vessel fMRI signals in the hippocampus. It is also noteworthy that the strong BOLD signal from the draining veins through the lateral ventricle can be distinguished from the hippocampal vasculature, showing a spatially more refined hemodynamic mapping than the EPI-fMRI mapping in Fig. 1f.

### Optogenetic single-vessel fMRI with concurrent Ca^2+^ recording

**Fig. 3:**
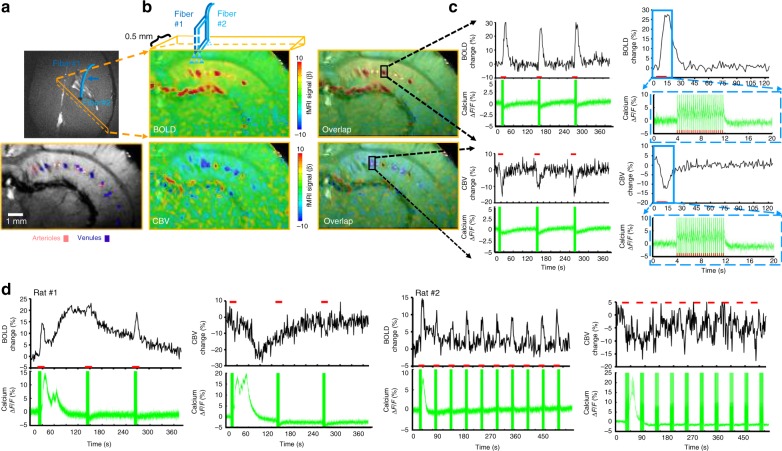
Concurrent fMRI and Ca^2+^ recording in the hippocampus. **a** Schematic drawing of the hippocampal single-vessel fMRI with two optical fibers (blue arrow) for optogenetic stimulation and Ca^2+^ recordings. A representative A–V map shows individual arterioles (bright dots, red markers) and venules (dark dots, purple markers) on the same 2D slice. **b** Evoked BOLD (upper) and CBV-weighted (lower) fMRI maps and overlapping maps on the A–V map. **c** Time courses of evoked BOLD and CBV-weighted fMRI signal from a single venule (upper) or arteriole (lower) ROI with the concurrent neuronal Ca^2+^ signal (illumination: 10 ms light pulse, 3 Hz, 8 s, 5 mW). Averaged time course of the fMRI signal and the evoked Ca^2+^ spike train. **d** A representative time course of single-vessel BOLD and CBV-weighted fMRI signal changes with concurrent hippocampus SDL-Ca^2+^ responses (illumination: 10 ms light pulse, 3 Hz, 8 s, 5 mW)

We performed a simultaneous bSSFP-based single-vessel optogenetic fMRI and optical fiber Ca^2+^ recording. Both optical fibers were inserted to target the CA1 region, and the 2D bSSFP slice was chosen to be 500 µm away from the optical fiber along the caudal–ventral axis (Fig. 3a), which avoided the potential focal vascular blood flow regulation by direct light exposure^48^. Upon optogenetic activation, both BOLD and CBV-weighted fMRI signals were detected from individual hippocampal venules and arterioles with concurrent Ca^2+^ transients following each light pulse (Fig. 3a–c), showing highly correlated NVC events in the hippocampus. Similar to the previous experiment, averaged time courses of evoked BOLD and CBV-weighted signals showed robust responses with altered amplitudes and durations from individual venules and arterioles at varying durations (1, 2, 4, and 8 s) and light pulse frequencies (1, 2, 3, and 5 Hz), which were detected simultaneously with evoked neuronal Ca^2+^ transients in the hippocampus (Supplementary Fig. 5). These results demonstrate the feasibility of multi-modal imaging of NVC events in the hippocampus, linking the evoked Ca^2+^ transients from CA1 neuronal ensembles to the widely spread vessel-specific hemodynamic responses in the sub-centimeter scale hippocampal vasculature.

In contrast to the trains of Ca^2+^ transients evoked by low-frequency light pulses, the evoked Ca^2+^ signal did not return to baseline between light pulses at a higher frequency, showing an accumulative Ca^2+^ plateau response corresponding to the high-amplitude fMRI signal detected in hippocampal vessels (Fig. 3c, Supplementary Fig. 5). Interestingly, we also observed a large-scale hippocampal Ca^2+^ transient, that is, the SDL-Ca^2+^ transient, at inter-stimulus intervals following optogenetic stimulation with 3 or 5 Hz light pulses at 8 s stimulation-on duration (Fig. 3d). The SDL-Ca^2+^ transients coincided with the spreading positive BOLD and negative CBV-weighted signals during inter-stimulus intervals in the hippocampus (Fig. 3d).

**Fig. 4:**
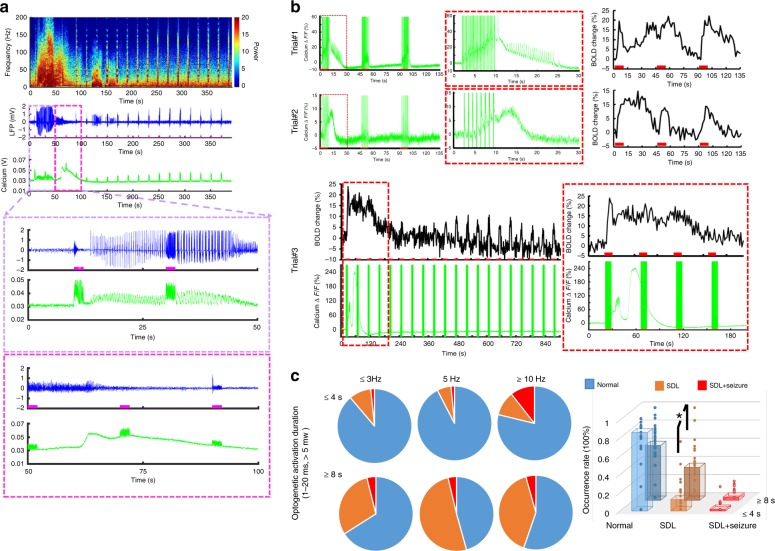
SDL and seizure-based Ca^2+^ recordings with LFP or fMRI. **a** Representative traces of SD and seizure spikes in the hippocampus: power spectra of LFP (top), LFP trace (middle), and Ca^2+^ signal (bottom). Zoomed views of the area are outlined in the upper panel (purple/pink box) (illumination: 10 ms light pulse, 50 Hz, 2 s, 5.5 mW). **b** Representative trials (3 rats) of neuronal Ca^2+^ signal (green) with simultaneous single-vessel BOLD fMRI signal (black) (upper: 10 ms light pulse, 1 Hz, SDL and 3 Hz, SDL + seizure Ca^2+^ events, 8 s, 6 mW; lower: 20 ms light pulse, 5 Hz, 8 s, 5.5 mW). Zoomed views of the area are outlined in the red box. **c** Quantification of the occurrence percentage of normal, SDL and SDL + seizure Ca^2+^ events as a function of optogenetic light pulse frequency (≤3 Hz, 5 Hz, ≥10 Hz) and stimulation duration ((≤4 s, 382 trials, ≥8 Hz, 448 trials, *n* = 28 rats). Induction rate of SDL-Ca^2+^ events in trials with 8 s stimulation duration is significantly higher than that of trials with 4 s stimulation (*p = 0.001, *n* = 28 rats, mean ± SEM)

As previously reported^52,53^, the high-frequency optogenetic activation (>10 Hz) in the hippocampus leads to seizure-like events in animals. The simultaneous LFP and fiber optic Ca^2+^ recordings also detected epileptic events as a train of strong LFP deflections and concurrent Ca^2+^ transients. These epileptic events were often accompanied by a large amplitude SDL-Ca^2+^ event in the hippocampus (Fig. 4a), which was previously reported in the cortex of both animal and human brains^54,55^, but not with concurrent fMRI. Interestingly, the epileptic Ca^2+^ transients could be elicited concurrently with SDL-Ca^2+^ during inter-stimulus intervals even with 3 and 5 Hz light pulse stimulation, followed by the spreading positive BOLD signal from individual vessels in the hippocampus (Fig. 4b). Occasionally, after spontaneous high-amplitude Ca^2+^ events, evoked single-vessel BOLD signals were diminished in the following 5–6 min and then gradually recovered with reduced amplitude, indicating the conventional depression pattern (Fig. 4b, trial #3). We have systematically analyzed occurrence rates of the SDL and SDL with seizure (SDL + seizure) events across multiple trials recorded from rats, showing that the occurrence rate is dependent on the optogenetic light pulse frequency and stimulation duration (Fig. 4c). When the light pulse stimulation duration is longer than 8 s, the occurrence rate of the SDL events (36.2 ± 5.5%) was significantly higher than that (12.5 ± 3.8%, *p* = 0.001) of the 4 s stimulation-on duration (Fig. 4c). These results demonstrate that the multi-modal fMRI platform can detect both optogenetically evoked and spontaneous SDL-Ca^2+^ transients with specifically coupled fMRI signals, presenting a unique scheme to investigate the NVCe to varied hippocampal activities.

### Vessel-specific NVCe at different forms of Ca^2+^ spikes

**Fig. 5:**
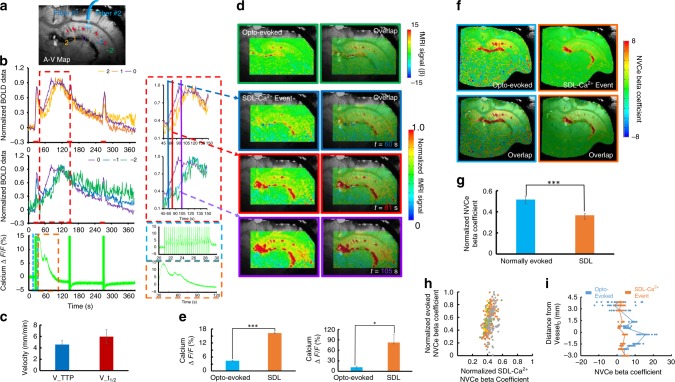
Ca^2+^-based NVCe mapping. **a** Numbered venules on the A–V map. Venule 0 (purple) is the one closest to the optical fiber tip, venules 1, 2 and −1, −2 are vessels with different directions in the hippocampal structure. **b** Time courses of normalized single-vessel BOLD fMRI signal from individual venules, as shown in Fig. 4a, are plotted (top) with the concurrent neuronal Ca^2+^ signal (bottom). Insets are the magnified figures of the dashed box in the left to highlight the SDL-Ca^2+^ events and coupled single-vessel BOLD responses (illumination: 10 ms light pulse, 3 Hz, 8 s, 5 mW). **c** Velocity of SDL-Ca^2+^ events spreading between different hippocampal venules with different time to peak (TTP) and *t*_1/2_ (18 vessels from 3 rats, mean ± SEM). **d** Representative color-coded BOLD fMRI map (optogenetically evoked) from the hippocampus (upper). Time-lapsed function maps and semi-transparent overlapping images on the A–V map at 60 s (blue box), 81 s (red box), and 105 s (purple box) during the trial. **e** Amplitude of optogenetically evoked Ca^2+^ signals is significantly lower than that of SDL-Ca^2+^ spikes in the hippocampus (left: *p = 6.22e − 11, 10 trials from a representative rat; right: *p = 0.028, *n* = 4 rats). **f** NVCe coefficient map for both optogenetically evoked and SDL-Ca^2+^ events. **g** *Z*-score normalized NVCe coefficients of optogenetically evoked events are significantly higher than those of SDL-Ca^2+^ events (*n* = 4 rats, *p = 0.003). **h** Scatter plot of *z*-score normalized NVCe coefficients from optogenetically evoked vs. SDL-Ca^2+^ events from each trial in four representative rats. **i** *Z*-score normalized NVCe coefficients from individual vessels were plotted as a function of vessel distance for both optogenetically evoked and SDL-Ca^2+^ events in a representative rat

Different from the random incidence of epileptic events (Fig. 4c, ≤4 s, 2 ± 1.1%, ≥8 s, 3.5 ± 1.2%), SDL-Ca^2+^ events were often detected after the first epoch of 8 s optogenetic stimulation across multiple trials of several animals (≥50% induction rate, Fig. 4c, 8 s, 5 Hz, and ≥10 Hz conditions). It enabled to statistically compare the spatiotemporal hemodynamic response pattern and the NVCe between the SDL and the optogenetically evoked Ca^2+^ transients in the hippocampal vasculature. The bSSFP-fMRI method was used to characterize the distinct spatiotemporal hemodynamic patterns of the single-vessel fMRI signal coupled to either optogenetically evoked or SDL-Ca^2+^ events in the hippocampus. The single-vessel hippocampal A–V map can be used to specify the relative position of individual vessels with respect to the optical fiber tip (Fig. 5a). First, BOLD fMRI signals from individual venules were extracted to show the temporal dynamics corresponding to different Ca^2+^ events. In contrast to the evoked BOLD signals that co-occurred instantaneously across different hippocampal penetrating venules upon the optogenetic stimulation, the SDL-Ca^2+^-coupled BOLD signals presented a propagation delay from individual venules as a function of distance to the optical fiber. Figure 5b shows the early onset and time to peak (TTP) from the venule closest to the optical fiber (*V*_0_), and the delayed onset time and TTP from hippocampal venules aligned further away from the optical fiber (*V*_−1,−2_, *V*_1,2_). BOLD signal propagation velocity was estimated by measuring TTP or the half-peak onset time (*t*_1/2_) across different hippocampal venules (*v*_TTP_ = 4.58 ± 0.47 mm/min; $v_{t_{1/2}}$ = 5.94 ± 1.31 mm/min) (Fig. 5c), which fell into the top-end SD propagation speed range (1–6 mm/s) detected in the cortex^54–56^. In addition, time-lapsed fMRI maps show slowly spreading BOLD signals from individual venules through the sub-centimeter scale hippocampal vasculature corresponding to SDL-Ca^2+^ events, which are different from the optogenetic activation pattern specific to evoked Ca^2+^ events (Fig. 5d, Supplementary Movie 4). Also noteworthy is the fact that although peak BOLD amplitudes were similar between the two different forms of Ca^2+^ events, the evoked Ca^2+^ transient amplitude was significantly lower than that of the SDL-Ca^2+^ event across different trials from the same animal and among different animals (Fig. 5e). These results suggested that varied NVC events are coupled to the optogenetically evoked and SDL-Ca^2+^ signal, and can be directly measured with the multi-modal single-vessel fMRI method in the hippocampus from the same experimental trials.

Concurrent single-vessel fMRI and Ca^2+^ signals can be used to estimate the efficiency of the vessel-specific NVC according to the different forms of neuronal activity. In contrast to the conventional general linear model (GLM) that fits the fMRI signal with the ideal time course, describing the hemodynamic function derived from the stimulation paradigm, we analyzed the concurrent Ca^2+^ signal amplitude and applied an amplitude modulated (AM) response model to calculate *β*-coefficients, as estimates of the NVCe to the optogenetically evoked and SDL-Ca^2+^ events (Supplementary Fig. 6, see Methods section for details)^21^. Figure 5f demonstrates NVCe *β* maps of the two forms of Ca^2+^ events, showing peak NVCe *β* values on individual hippocampal venules. Mean vessel-specific NVCe *β* values of optogenetically evoked Ca^2+^ events were significantly higher than those of SDL-Ca^2+^ events (Fig. 5g). To better quantify the spatial distribution of the NVCe across hippocampal vasculature, we plotted *β*-coefficients from individual venules as a function of the relative distance to *V*_0_ (Fig. 5i). Despite the fact that the SDL-Ca^2+^ events were elicited in the hippocampal structure close to *V*_0_, the NVCe *β* values were found to be similar and evenly distributed across the hippocampal vasculature, whereas NVCe *β* values of optogenetically evoked Ca^2+^ events showed a distance-dependent distribution (Fig. 5h, i, scatter plot of NVCe *β* values from all hippocampal vessels through multiple trials of four animals). These results demonstrate altered NVCe linking to normal and SDL hippocampal activity detected by the multi-modal fMRI platform.

## Discussion

Here, we developed a multi-modal fMRI platform to investigate detailed, spatiotemporally resolved NVC events in rat hippocampus. By implementing simultaneous optogenetic single-vessel fMRI and optical fiber Ca^2+^ recordings, distinct hemodynamic spatiotemporal patterns across the sub-centimeter hippocampal vasculature could be directly characterized based on concurrent neuronal Ca^2+^ signals, for example, optogenetically evoked or SDL-Ca^2+^ events, for the first time. We believe that this method provides a unique multi-modal/cross-scale mapping scheme for the study of neurovascular activity in the hippocampus in both normal and pathological conditions.

Despite extensive imaging studies on hippocampal neural activity, the actual information flow from neuronal activity to the hippocampal neurovascular system, the modulation of which provides the vast majority of fMRI signals, has seldom been taken into account in investigations attempting to relate behavior to the function or dysfunction of this structure^30,33,57^. One major barrier is our ability to access large-scale hippocampal vascular dynamics in vivo with minimally invasive procedures, preserving NVC function. Three critical features needed to be solved for existing neuroimaging methods: large field of view (FOV), high resolution to detect the vessel-specific hemodynamic signal with sufficient signal-to-noise ratio (SNR), and accessibility to deep brain nuclei. Although wide-field two-photon or the newly developed three-photon microscopy has significantly enlarged the FOV and the penetration depth for in vivo brain optical imaging with cellular resolution^39–41,58^, it remains challenging to acquire vascular hemodynamic signaling through the sub-centimeter hippocampal structure in rats and higher mammals.

The fMRI signal directly represents vascular hemodynamic responses to indicate large-scale brain function. Our work and other animal fMRI studies have demonstrated the optogenetically evoked hippocampal BOLD signal in the context of whole-brain functional mapping^52,59^. By improving the spatial resolution of the fMRI image, it is possible to detect fMRI signals from individual penetrating vessels through the cortex^17,18,60,61^. The real power of single-vessel fMRI can be further released when targeting large deep brain regions beyond the penetration depth of conventional optical methods. To achieve sufficient SNR, the bSSFP-based single-vessel fMRI method was applied with an implanted surface RF coil^22^. The RF coil implantation could be merged with the optical fiber targeting the hippocampus during the surgical procedure. The RF coil implanted to the skull substantially increased *B*1 field sensitivity and prevented additional signal loss due to the extra space occupied for fiber fixation between the surface coil and the brain, which can be readily implemented by MRI users with 7 to 11.7 T scanners. This optimized multi-modal fMRI platform employed optogenetic single-vessel fMRI mapping to detect venule-specific BOLD and arteriole-specific CBV-weighted signals from individual vessels aligned in parallel through the hippocampal structure (Fig. 2), which have been previously described only by histological studies^42,43^. We delivered optical fiber-mediated optogenetic stimulation using light pulses with varied frequencies and power levels to specify BOLD and CBV-weighted fMRI signals from individual hippocampal vessels (Supplementary Fig. 4), as well as evoked LFP spikes and Ca^2+^ transients (Supplementary Fig. 1), representing highly correlated NVC features in the hippocampus. Single-vessel fMRI provides a unique mapping scheme to identify large-spread hemodynamic response patterns in the hippocampal vasculature.

Optogenetic light exposure may contribute directly to hemodynamic responses in the hippocampus. As reported by Rungta et al.^48^, direct light exposure can directly regulate blood flow through arteriole dilation following the reduced Ca^2+^ signal from smooth muscle cells, similar to NVC events but independent of neuroglial activity. Our work and a previous optogenetic fMRI study^49^ on naive animals mainly detect the susceptibility-based non-physiological MRI signal changes sensitive to the light-induced heating effect, which can shadow the MRI signal relevant to blood flow changes as detected by ultrasound Doppler measurement. In addition, the direct heating effect through light illumination can alter the spiking activity in the animal brain^62^. To avoid the confounding effects of light exposure-induced blood flow regulation, we have aligned the single-vessel 2D slice at least 500 µm away from the optical fiber tip. Also, both two-photon and single-vessel fMRI studies have shown that the spatial scale of the hemodynamic coherence in the arteriole network is <2 mm spatial scale in the cortex^22,58^. The spatial distribution of light exposure-induced flow regulation can be controlled using light pulses with low frequency and power, which could further reduce direct light-exposure contribution to the sub-centimeter scale hippocampal vascular hemodynamic responses and heating-induced neuronal activity modulation.

Using GCaMP6f, optogenetically evoked hippocampal neuronal Ca^2+^ transients showed similar temporal dynamics to cortical Ca^2+^ transients detected by the optical fiber in anesthetized rats upon sensory stimulation^21^. This temporal feature makes it possible to distinguish individual Ca^2+^ transients from light pulse-induced artifacts, which has been previously reported in the Opto-fMRI with Ca^2+^ dye (OGB-1) sensing-based optical fiber measurements^28^. To reduce the spectral signal cross-talk, we applied a red-shifted ChR2 variant (C1V1) to alter the optogenetic light pulse up to 590 nm (Supplementary Fig. 3). Although the artifacts can be significantly reduced, the remaining light pulse signals detected by the photomultiplier can be caused by imperfect filtering of the dichroic filter. It is noteworthy that the optogenetic light pulses were delivered at 1–5 mW from the 200 µm optical fiber tip, which is significantly higher than the power used for GCaMP fluorescent excitation (5–10 µW)^21^. Since the optical excitation was delivered at a low power level for continuous Ca^2+^ signal recording, its effect on optogenetic activation is negligible.

To better validate hippocampal Ca^2+^ transients free of optogenetic light pulse artifacts, we also observed individual Ca^2+^ transients coinciding with interictal LFP spikes during seizure induction following high-frequency optogenetic activation (Fig. 4). These interictal spikes paired with the train of spontaneous Ca^2+^ transients have also been observed in the mouse cortex with an optogenetically induced seizure^63^. Also, we observed robust SDL-Ca^2+^ events during inter-stimulus intervals in the hippocampus following 8 s (≥5 Hz) optogenetic stimulation (Fig. 4). These SDL-Ca^2+^ events were recently reported to follow trains of interictal spikes during the optogenetically induced seizure^63^ in the mouse cortex, which could be reliably detected in the hippocampus following optogenetic stimulation with fMRI. In our study, the number of seizure events detected by calcium recordings is much smaller than that of the SDL events, which might be due to the lack of sensitivity of calcium recording to detect interictal spikes through the 8-m optical fiber. The multi-modal fMRI mapping scheme allows us to specify unique NVC patterns according to different formats of hippocampal activity.

The hippocampal CA1 region has been considered as a key component in the framework of epilepsy induction and treatment^53,57,64^. Previous hippocampal Opto-fMRI studies have also shown the seizure behavior in animals following high-frequency light exposure, demonstrating broad hippocampal BOLD fMRI spatial patterns and global hemodynamic effects concurrent with epileptic events^52,65^, which are different from the evoked hippocampal activity with Opto-fMRI^59,66^. Epileptic events observed in the cortex are usually accompanied by cortical spreading depression^54,55,63^, which is typically studied with fMRI by direct KCl topical treatment or focal ischemia in animal brains^67^. In the hippocampus, we detected robust SDL-Ca^2+^ events independent of epileptic activity in inter-stimulus intervals when 3–5 Hz optogenetic light pulses were used (Fig. 3d, Supplementary Fig. 5B), which have dominated the random incidence of epileptic events using similar stimulation paradigm in the hippocampus. The optogenetically induced cortical spreading depression has been reported in the mouse cortex without seizure induction^68^. These SDL-Ca^2+^ events link to specific spatiotemporal patterns of hippocampal vascular hemodynamic responses. Intermediate characteristics of SDL-Ca^2+^-specific NVC events can be quantitatively examined to bridge the normal condition to the typical spreading depression, as well as hippocampal epileptic events despite their scarce occurrence in normal animals.

Coupled to SDL-Ca^2+^ events, the BOLD signal propagation through individual hippocampal vessels has a 4–6 mm/min velocity in a ~6–8 mm spatial scale (Fig. 5c). This speed falls to the relatively top-end propagation speed range of cortical SD detected from the neuronal network^54–56,68,69^. This observation might be because of intrinsic architectural differences between the cortex and the hippocampus; the latter is highly susceptible to SD and may sustain faster SD propagation speed^70,71^. Kunkler et al.^72^ have observed Ca^2+^ waves occurring in neurons (~6 mm/min) and astrocytes (~4 mm/min) during SD initiation and propagation in hippocampal organ cultures. Meanwhile, in contrast to astrocytic Ca^2+^ waves propagation speed of (2–3.3 mm/min) related to cortical SD in both rat and mouse neocortex^69,73,74^, Heuser et al.^75^ also reported a 6–8 mm/min propagation speed of SD Ca^2+^ waves from both neurons and astrocytes in the hippocampal CA1 region. Interestingly, unique, spontaneous astrocytic Ca^2+^ waves, which have been reported to propagate at ~4 mm/min in the hippocampus, do not show the long-term spreading depression features^76^. It is noteworthy that the neuronal Ca^2+^ signal was only acquired through the single optical fiber inserted into the hippocampus with limited coverage of neuronal activation in comparison to the large-scale hippocampal vascular dynamic mapping with single-vessel fMRI. To better characterize neuron–glial–vascular interaction at various brain states in the hippocampus, we will apply the multiple fiber insertion with single-vessel fMRI to image transgenic mice expressing GCaMP specifically in astrocytes^77^, as well as the right-shifted calcium indicator in neurons^78^, with a multi-slice single-vessel fMRI method to cover the three-dimensional hippocampal structure.

Besides the similarity of the propagation speed of SDL-Ca^2+^-specific hemodynamic responses to SD events reported in the hippocampus, the baseline Ca^2+^ signal was reduced following the SDL-Ca^2+^ event for 5–6 min (Fig. 3d). Nevertheless, we only detected the correlated BOLD signal increase and CBV-weighted signal decrease (due to vasodilation), but no clear sign of vasoconstriction-based fMRI signal change was detected. Also, suppressed fMRI signals recovered in 3–6 min in most of the SDL-Ca^2+^ events (except for one dramatic case showing a long-term depression over 10 min, which was shown in Fig. 4b). Interestingly, although no clear vasoconstriction-based fMRI signal was detected following the SDL-Ca^2+^ event, significantly reduced NVCe was detected when comparing to the optogenetically evoked Ca^2+^ transients (Fig. 5). Consistent with impaired NVC during cortical SD^54,79^, we provide a multi-modal fMRI platform to directly measure altered NVCe directly linked to concurrent SDL-Ca^2+^ events in the hippocampus.

In summary, we have developed a multi-modal fMRI platform to acquire concurrent neuronal Ca^2+^ and single-vessel fMRI signal in a subcortical brain region, for example, the hippocampus. This method allows for detecting hemodynamic fMRI responses from individual vessels through the sub-centimeter hippocampal vasculature. In particular, when neuronal activation is elicited in the hippocampus, large-scale vascular hemodynamic responses can be represented based on the estimated NVCe. This multi-modal fMRI platform will possibly be used to specify distinct NVC events through the hippocampal structure in animal models at various disease states.

## Methods

### Animal preparation and instrument setup

All surgical and experimental procedures reported in this paper were approved by local authorities (Regierungspraesidium, Tübingen Referat 35, Veterinärwesen, Leiter Dr. Maas) and were in full compliance with guidelines of the European Community (EUVD 86/609/EEC) for the care and use of laboratory animals. Experimental animals were rodents, specifically Sprague–Dawley male rats, ~3 to 4 weeks old, or ~90 g, provided by Charles River Laboratories in Germany. The rats were housed in transparent Plexiglass cages (381 × 513 × 256 mm^3^) under conditions of well-controlled humidity and temperature. A 12–12 h on/off lighting cycle was maintained to assure an undisturbed circadian rhythm. Food and water were obtainable ad libitum. A total of 37 male Sprague–Dawley rats were used at 2–3 months of age. Five rats were imaged under 14 T at the Max Planck Institute (both BOLD and CBV-weighted fMRI data with A–V maps were acquired from four of five rats). In 12 rats, optogenetically driven neuronal calcium signals were concurrently recorded with BOLD/CBV-weighted signals (in 4 of these 12 rats optogenetically evoked responses and SDL-Ca^2+^ events were acquired). Twenty rats were used for concurrent optogenetically driven electrophysiological and neuronal calcium recordings. For the SDL and seizure induction rate calculation, 4 of the 32 rats were not included due to a failed calcium signal detection from hippocampal neurons. If the optical fiber insertion caused severe micro-bleeding in the hippocampus, which led to poor calcium recording and optogenetic activation, the data acquired from that rat was not included in the statistical analysis.

### Viral injection

After 1 week habitation, rats were injected with the non-replicating AAV vectors into BC and hippocampus (AAV5.Syn.GCaMP6f.WPRE.SV40: Addgene100837-AAV5; AAV5.CAG.hChR2 (H134R)-mCherry.WPRE.SV40: Addgene100054-AAV5; AAV9-CaMKIIa-C1V1 (t/t)-TS-EYFP: Addgene35499-AAV9). Viral vectors were procured from the University of Pennsylvania Vector Core. The injection process was carried out under isoflurane anesthesia with an induction concentration of 5.0% and a maintenance concentration of 1.5–2.0% in an oxygen-enhanced gas (30% oxygen). Following their stabilization, the rats were secured in a stereotaxic apparatus (Model 900, David Kopf Instruments). Eyes of the rats were protected with an ophthalmic ointment (Puralube), and the level of anesthesia was regularly checked by testing toe and tail pinch reflexes. With a midline incision, one small craniotomy (1–2 mm) was performed above the region of interest by using a dissecting microscope (Leica) and a pneumatic drill (Ideal Micro Drill, Harvard Apparatus). A 10 μL syringe (NanoFil, World Precision Instruments Inc.) with a 35 gauge beveled metal needle (World Precision Instruments Inc.) were placed in the stereotactic frame and slowly lowered towards target sites (BC: caudal, 2.5 mm, lateral, 5.0 mm, and ventral, 1.5~0.9 mm; hippocampus: caudal, 4.2 mm, lateral, 2.8 mm, and ventral, 2.75–2.65 mm, respectively, from bregma). The flow rate of the virus injection was controlled by an infusion pump (Pump11 Elite, Harvard Apparatus) at a speed of 0.1 μL/min. The total injection volume was around 0.2–0.6 μL (ChR2)/0.6–1 μL (GCaMP6f). After the injection, the syringe needle was kept in place for an additional 10 min before being slowly withdrawn. The hole was sealed by bone wax (W31G, Ethicon), and the incision was sutured. Ketoprofen [5 mg/kg, q.d. (once/day)] was subcutaneously injected to relieve postoperative pain for 3 days after surgery. fMRI experiments were performed 4–8 weeks after the injection to ensure the expression of the AAV viral vectors.

### Optical fiber/electrode preparation and implantation

Optical stimulation and electrophysiological recordings were performed with a 2 m (bench experiment) or 8 m (fMRI experiment) optic fiber (FT200-EMT, NA = 0.39, 200 μm, Thorlabs). The coating of both ends of the optical fiber was stripped off. One end was glued into an FC/PC connector (Thorlabs), and the other end was carefully polished by using polishing sand papers with appropriately selected grit size (LF1P/3P/5P, Thorlabs). Optical quality of the polishing interface was confirmed by using a fiber inspection microscope (FS200, Thorlabs). For simultaneous Ca^2+^ recording and fMRI, two fibers (one for optical stimulation, the other for Ca^2+^ recording) were closely glued (454, Loctite) together. For simultaneous Ca^2+^ and electrophysiological recording, a Tungsten electrode (1 MΩ, ~100 μm, FHC) was closely glued to the optical fiber tips. The dura was carefully removed, the optical fibers with the electrode were slowly inserted into either the BC or the hippocampus. The reference and ground were placed on the screws, which were fixed above the cerebellum. After implantation, the fibers with the electrode were glued to the skull for acute terminal experiments.

### Animal preparation for fMRI

The experiments were described in the previous studies^18,45^. Briefly, after induction of anesthesia, animals were endotracheally intubated with a mechanical ventilator (SAR-830, CWE). Plastic catheters (PE50, INSTECH) were carefully implanted into the right femoral artery/vein of rats to administer drugs and monitor arterial blood gases. After catheterization, the rats were secured into a stereotaxic apparatus. One small craniotomy (1–2 mm) was performed just above the regions of the virus injection and the dura was carefully removed. Two optical fibers were slowly inserted into the virus expression regions in the hippocampus, and the fibers together with a custom-made coil were fixed above the skull by using super glue (454, Loctite). Around 30 min for fixation, after the injection of a bolus of α-chloralose (60 mg/kg, intravenously (i.v.)), the rats were transferred into the MR scanner (14T, Bruker). Maintenance anesthesia was switched from isoflurane to continuous infusion of α-chloralose (infusion rate: 26.5 mg/kg/h). Throughout the whole experiment, the rectal temperature of the rat was monitored and maintained at around 37 °C by using a feedback heating system. All relevant physiological parameters were continuously monitored, including rectal temperature, arterial blood pressure (Biopac 150, Biopac Systems Inc.), pressure of the tidal ventilation (SAR-830, CWE), and end-tidal CO_2_ (capnometer, Novametrix). Arterial blood gas was measured regularly to guide physiological status adjustments by changing the respiratory volume or administering the sodium bicarbonate (8.4%, Braun) to maintain normal pH levels. For α-chloralose anesthetized animals, a muscle relaxant (pancuronium bromide, 1 mg/kg/h) was intravenously injected to minimize motion artifacts. Dextran-coated iron oxide (15 mg of Fe/kg, BioPAL, MA, i.v.) was additionally injected for obtaining high SNR CBV-weighted signal.

### Optogenetic-driven Ca^2+^ with electrophysiological recording

Around 4–8 weeks after virus injection, LFP signal and Ca^2+^ signal were simultaneously recorded in a terminal experiment. Virus injection coordinates were first confirmed by a FLASH (fast low angle shot) anatomical MRI image before surgery. Anesthetics and surgical preparation procedures were similar to those of the fMRI experiments. After insertion, the LFP signal was amplified using a BioPac EEG100C module (gain factor, 5000, bandpass filter, 0.02–100 Hz, sampling rate, 5000/s). The GCaMP6f-mediated Ca^2+^ signal was recorded by the analog input module of the BioPac 150 system. For electrical stimulation, electrodes were placed into rat whisker pads and later delivered electrical pulse sequences (1.0–2.0 mA, 330 μs duration repeated at 1–5 Hz) by using a high voltage stimulator (A360LA, WPI). Stimulation was controlled by the Master-9 AMPI system (Jerusalem, Israel) based on the stimulus paradigm, and triggering pulses were recorded by the analog input module of the BioPac 150 system (sampling rate, 5000/s). For optical stimulation, light pulses were delivered through the 473 nm laser (CNI, China). An analog module was applied to trigger the light pulse to give the optical stimulation with different pulse durations (1, 2, 4, and 8 s). Light intensity from the fiber tip was measured by using fiber optical power meters (PM20A, Thorlabs), which were controlled from 1 to 36 mW (light power higher than 40 mW was beyond the measurement range).

### Perfusion, section, and microscope

In all terminal experiments, after completion of the data acquisition, rats were euthanized under deep anesthesia with isoflurane (5%). They were subsequently transcardially perfused with 0.1 M ice-cold phosphate-buffered saline (PBS, Gibco) and 4% paraformaldehyde (PFA). Brains were carefully removed from the skulls and placed into 4% PFA for post fixation (4 °C, overnight). Then, they were cryoprotected in 30% sucrose in PBS at 4 °C for 2–3 days before being flash frozen in OCT on dry ice and finally stored at −80 °C. Brain slices were sectioned in 30 μm thickness using a cryostat (CM1860, Leica). Brain slices were mounted on glass slides (Super-frost, Fisherbrand) and covered with coverslips. A mounting medium with DAPI (4′,6-diamidino-2-phenylindole; VectaShield, Vector) was used to protect the fluorescence signal and to stain nuclei. Wide-field images were acquired to assess the expression of ChR2/GCaMP in the BC and the hippocampus with a microscope (Zeiss). The images were minimally processed by ImageJ to enhance the brightness for visualization purposes.

### The optical setup for optical fiber Ca^2+^ recordings

The light path was built based on a previous report (Fig. 1a)^21,22^. The light source comes from a 488 nm laser (MBL-III, CNI). Light beams were first reflected through a dichroic mirror (F48-487, reflection 471–491 nm, >94%, transmission 500–1200 nm, >93%, AHF). Then, by using an objective lens fixed on the fiber launch (MBT613D/M, Thorlabs), the light beam was focused on the optical fiber (FT200-EMT, NA = 0.39, 200 μm, Thorlabs). The laser intensity was measured at the optical fiber tip (5 µW for neuronal calcium recording) by an optical power meter (PM20A, Thorlabs). The same optical fiber guided the emitted fluorescent signal back to the light path. The light beam was successively passed through a dichroic mirror and an optical filter (F37-516, 500–550 nm bandpass, AHF). By using a tube lens (AC254-030-A1-ML, Thorlabs), the GCaMP6-mediated fluorescent signal was coupled to a Peltier-cooled SiPM with a transimpedance preamplifier (MiniSM-10035-X08, SensL). Before being recorded by the analog input module of the Biopac 150 system, the signal from the photomultiplier was amplified by a voltage amplifier (DHPVA-100, Femto).

### MRI and fMRI procedures

All images were obtained by using a 14T/26 cm horizontal-bore magnet (Magnex Scientific) interfaced through the Bruker Avance III (Bruker). The scanner has a 12 cm Magnet gradient set with a strength of 100 Gauss per cm (G/cm), and a 150 μs rise time (Resonance Research Inc.). Home-made surface transceiver RF coils with an internal diameter of 7.5 and 21 mm, respectively, were used for fMRI image acquisition.

### Echo planar imaging fMRI

EPI image acquisition was preceded by FASTMAP shimming (i.e., measuring *B*0 field plots along projections instead of mapping whole imaging planes). By adjustments of echo spacing, symmetry, and setting up the *B*0 compensation, it considerably increases the speed and performance. By using the custom-made single surface coil, parameters of the 3D gradient echo sequence were as below: volume TR = 1.5 s; TE = 14 ms; bandwidth: 170 kHz; flip angle: 12°; matrix: 64 × 64 × 16; in-plane resolution: 300 × 300 μm^2^; slice thickness: 500 μm. The paradigm consisted of 360 dummy scans enabling the emergence of reaching the steady state, 10 pre-stimulation scans, 3 scans during stimulation, and 12 post-stimulation scans with 8 epochs for each run or 5 scans during stimulation and 25 post-stimulation scans for 10 epochs. For anatomical images, the RARE (rapid imaging with refocused echoes) sequence was implemented to acquire images with the same geometry of the fMRI images.

### Balanced steady-state free precession fMRI

The bSSFP-fMRI method was applied to acquire evoked fMRI signals using the following parameters: TR: 11.7 ms; TE: 5.85 ms; matrix: 128 × 128; FOV: 12.8 × 12.8 mm^2^; in-plane resolution: 100 × 100 μm^2^; flip angle: 22° (BOLD); TR: 10.4 ms; TE: 5.2 ms; matrix: 96 × 96; FOV: 12.8 × 12.8 mm^2^; in-plane resolution: 130 × 130 μm^2^; flip angle: 17° (CBV); slice thickness: 500 μm. The paradigm consisted of 300 dummy scans to reach the steady state, 25 pre-stimulation scans, 1 scan during stimulation, and 14 post-stimulation scans with a total of 8 epochs for each run, 1 scan during stimulation, and 29 post-stimulation scans with a total of 10 epochs for each run, and 1 scan during stimulation and 79 post-stimulation scans with a total of 3 epochs for each run. CBV-weighted fMRI signals were acquired after intravenous injection of dextran-coated iron oxide (BioPAL, MA, i.v.).

### Single-vessel MGE Imaging

For the detection of the individual arterioles and venules in rat hippocampus, a 2D MGE sequence was applied with the following parameters: TR: 50 ms; TE: 2.5, 5.5, 11.5, 14.5, 17.5, 17.5, 20.5, 23.5 ms; flip angle: 58°; matrix: 256 × 192; in-plane resolution: 67 × 67 μm^2^; slice thickness: 500 µm. The MGE images were averaged from the 2nd echo to the 5th/6th echo to get the A–V map (Fig. 2c).

### Data analysis and statistics

Preprocessing and analysis of functional imaging data were carried out by using the software package, Analysis of Functional NeuroImages (AFNI) (NIH, Bethesda, MD). Evoked calcium signals were processed in Matlab (MATLAB, MathWorks, USA).

For calcium data analysis, neuronal calcium signals were low-pass filtered (100 Hz) by zero-phase shift digital filtering. The relative percentage change of the calcium fluorescence (∆*F*/*F*) was defined as (*F* − *F*0)/*F*0, where *F*0 is the baseline fluorescent signal.

For the EPI-fMRI analysis, EPI images were aligned to anatomical datasets, which were registered to template images across the trials. Baselines of EPI images were normalized to 100 for multiple trials of block-design statistical analysis of EPI time courses.

For bSSFP-fMRI analysis, the tag-based registration method was used to register the single-vessel functional map with the A–V map. We normalized time courses of bSSFP-fMRI signals from SDL-Ca^2+^ events by scaling the maximum to 1 (Fig. 5b). GLM analysis was applied to estimate evoked and SDL-Ca^2+^ NVCe *β*-coefficients. *β* Estimates were used to indicate the amplitude of the BOLD and CBV-weighted responses in *β* maps. For the CBV percentage (%) map, the %CBV was estimated based on the equation: %CBV = ln(*S*_Fe-base_/*S*_stim_)/ln(*S*_Fe-pre_/*S*_Fe-base_). The *S*_Fe-base_ is the baseline level fMRI signal after iron oxide particle injection, and *S*_Fe-pre_ is the baseline level fMRI signal before iron oxide particle injection^51^.

For the analysis of fMRI signals recorded simultaneously with hippocampal SDL-Ca^2+^ transients, an AM response model based on an AFNI script was implemented to perform GLM analysis.

The AM response model is given by:

$$r_{\mathrm{AM1}}(t) = \mathop {\sum}\limits_{K = 1}^K {h\left( {t - \tau k} \right)a_k},$$

where *α*_*k*_ is the value of *k*th amplitude of hippocampal SDL Ca^2+^ transient. *h*(*t*) is the hemodynamic response function based on the *γ* variate function implemented by the AFNI BLOCK function:

$$h\left( t \right) = \mathop {\int}\limits_0^{{\it{\mathrm{min}}}\left( {t,L} \right)} {s^4e^{ - s}/[4^4e^{ - 4}} ]{\mathrm{d}}s,$$

where *L* is the duration of the response. A varied duration (*L*) was applied to test the goodness of fit for the general linear model with *t* statistics reported in Supplementary Fig. 6. Both NVCe *β*-coefficients were estimated simultaneously, using GLM analysis implemented in the AFNI 3dDeconvolve function. The response regressors are shown in the equation:

$$Y\left( t \right) = \beta _1h\left( t \right) + \beta _2r_{\mathrm{AM1}}\left( t \right) + {\it{\epsilon }},$$

where *β*_1_ is the optogenetically evoked coefficient and *β*_2_ is the SDL-Ca^2+^ coefficient. *ϵ* is the error term. Polynomial terms regressing the baseline drift are not shown. The calculated *β*-coefficient was represented in a voxel-wise manner, as a *β* map, which can be overlapped on the A–V map of the 2D hippocampal slice in Fig. 5f or Supplementary Fig. 6D.

Evoked NVCe *β*-coefficients and SDL-Ca^2+^ NVCe *β*-coefficients were normalized within each trial to have zero mean and unit variance. Coefficients were scaled to the range 0–1 using the minimal and maximal values (Fig. 5g, h). Student’s *t* test (two-tailed) was performed for group analysis, to compare the calcium Δ*F*/*F* (Fig. 5e) or the normalized NVCe *β*-coefficient (Fig. 5g) between optogenetically evoked responses and SDL-Ca^2+^ events in calcium and fMRI data. Also, one-way analysis of variance was performed to examine the goodness of fit for the hemodynamic function with varied duration. Data with error bars were displayed as means ± SEM. *P* values <0.05 were considered statistically significant. No blinding and randomization design was needed in this work.

### Reporting summary

Further information on research design is available in the Nature Research Reporting Summary linked to this article.

## Supplementary information

Supplementary Information

Reporting Summary

Description of Additional Supplementary Files

Supplementary Movie 1

Supplementary Movie 2

Supplementary Movie 3

Supplementary Movie 4

Source data

## Data Availability

Raw data can be provided upon email request to the corresponding author. Excel files are included for each quantitative plot included in main figures. Source data underlying Figs. 2f, 4c, 5c, e, g–i are provided as a Source Data file. The data presented in the figures and other summary level data are contained within the Supplementary Files. Further information on research design is available in the Nature Research Reporting Summary linked to this article.

## Code availability

AFNI software (NIH, USA) and Matlab (MATLAB, MathWorks, USA) were used to process fMRI and simultaneously acquired calcium signals, respectively. Relevant source codes can be downloaded through https://afni.nimh.nih.gov/afni/. Related image processing codes can be provided upon direct email request to the corresponding author.
