# Supplementary material for: Mapping optogenetically-driven single-vessel fMRI with concurrent neuronal calcium recordings in the rat hippocampus
Source: Nat Commun. 2019 Nov 20;10:5239. doi: 10.1038/s41467-019-12850-x (PMC6868210; doi:10.1038/s41467-019-12850-x)
Supplement: Supplementary file 1 — Supplementary Information [file 41467_2019_12850_MOESM1_ESM.pdf]

Supplementary information

Mapping optogenetically-driven single-vessel fMRI with concurrent neuronal calcium recordings in the rat hippocampus

Chen et al.

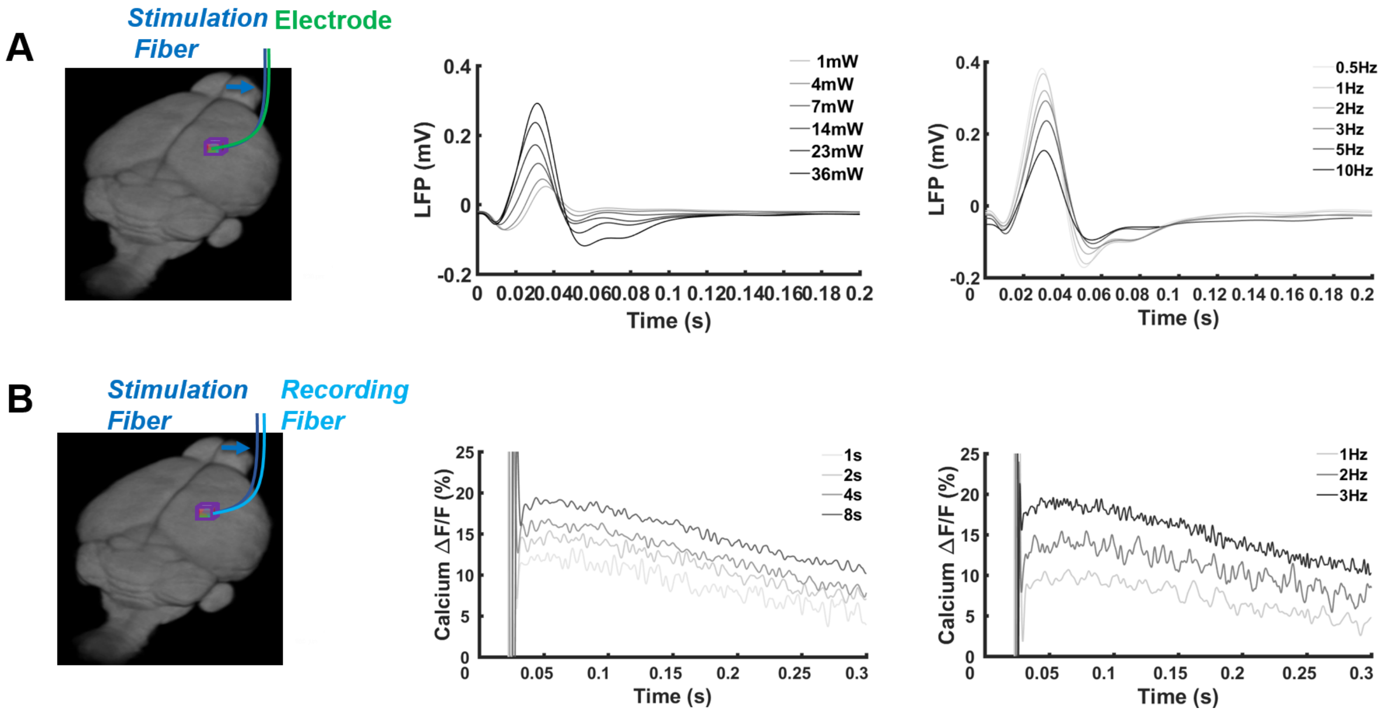

Supplementary Figure 1. Optogenetically evoked LFP and GCamp6f-mediated Ca^{2+} recording in the hippocampus.

A. Optogenetically evoked LFP traces with 10 ms light pulse stimulation at different powers (1-36 mW) averaged from trails with 3 Hz light pulse stimulation paradigm and frequencies (0.5-10 Hz) averaged from trails with 14 mW power of the light pulse (4 s on with 16 s off repeated for 20 times).

B. Optogenetically evoked neuronal Ca^{2+} traces with 10 ms light pulse stimulation at different stimulation on durations (1, 2, 4, 8 s) and frequencies (1-3 Hz) from experimental trails with each epoch in 20 s (1-4s on) and 45 s (8s on) repeated for 20 times.

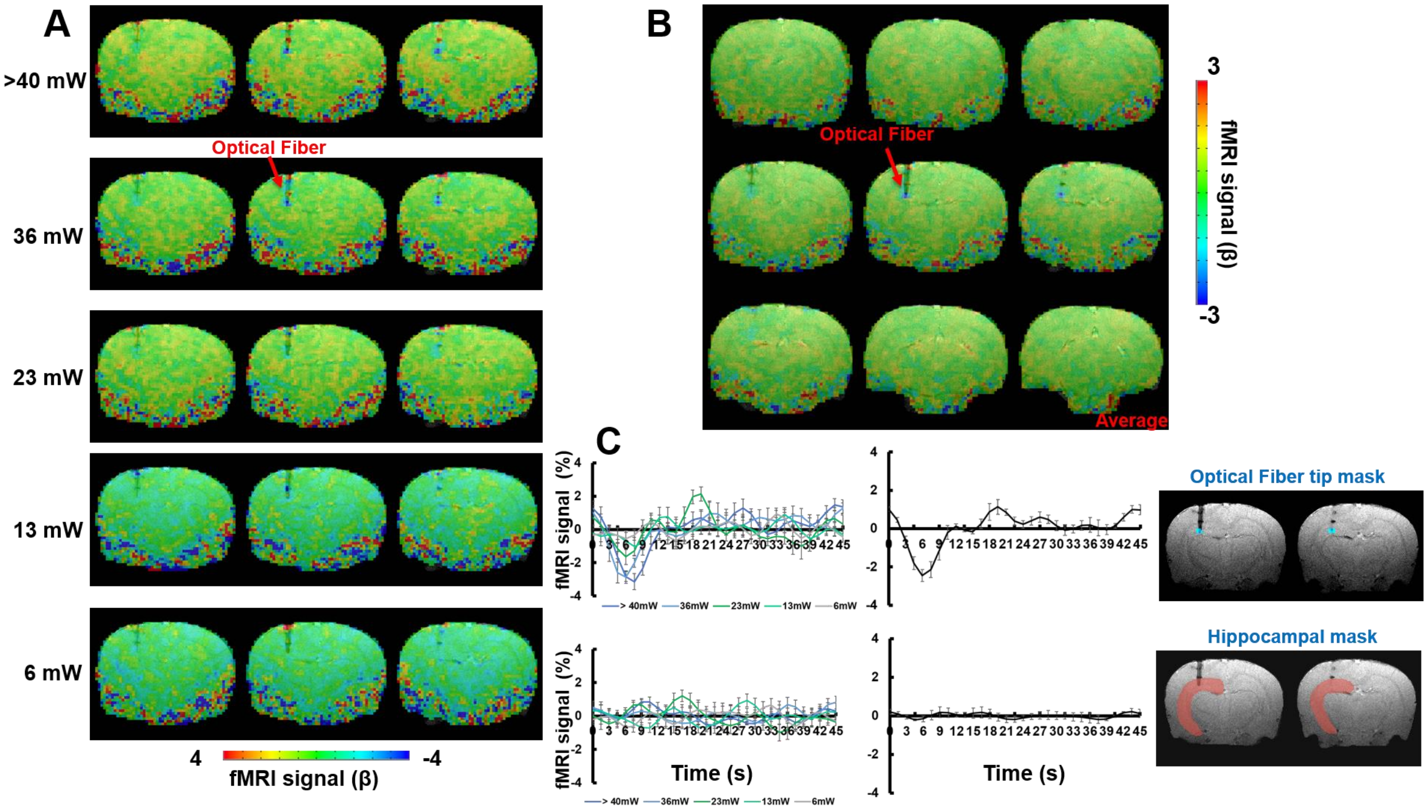

Supplementary Figure 2. Blue light-evoked B0 offset-induced MRI signal changes close to the fiber tip in the naive rat hippocampus.

A. Representative color-coded EPI-fMRI maps at different powers (6->40 mW) (illumination: 10 ms light pulse, 10 Hz, 8 s).

B. Averaged color-coded EPI-fMRI map from different powers (13, 23, 36, >40 mW) (illumination: 10 ms light pulse, 10 Hz, 8 s).

C. Averaged EPI-fMRI time courses from optical fiber tip (upper) and hippocampus (lower) at different stimulation powers (6->40 mW), averaged EPI-fMRI time courses from all the different powers (23, 36, >40 mW) (middle), and ROIs in blue contour (optic fiber tip) and red contour (hippocampus) of the MRI images (right).

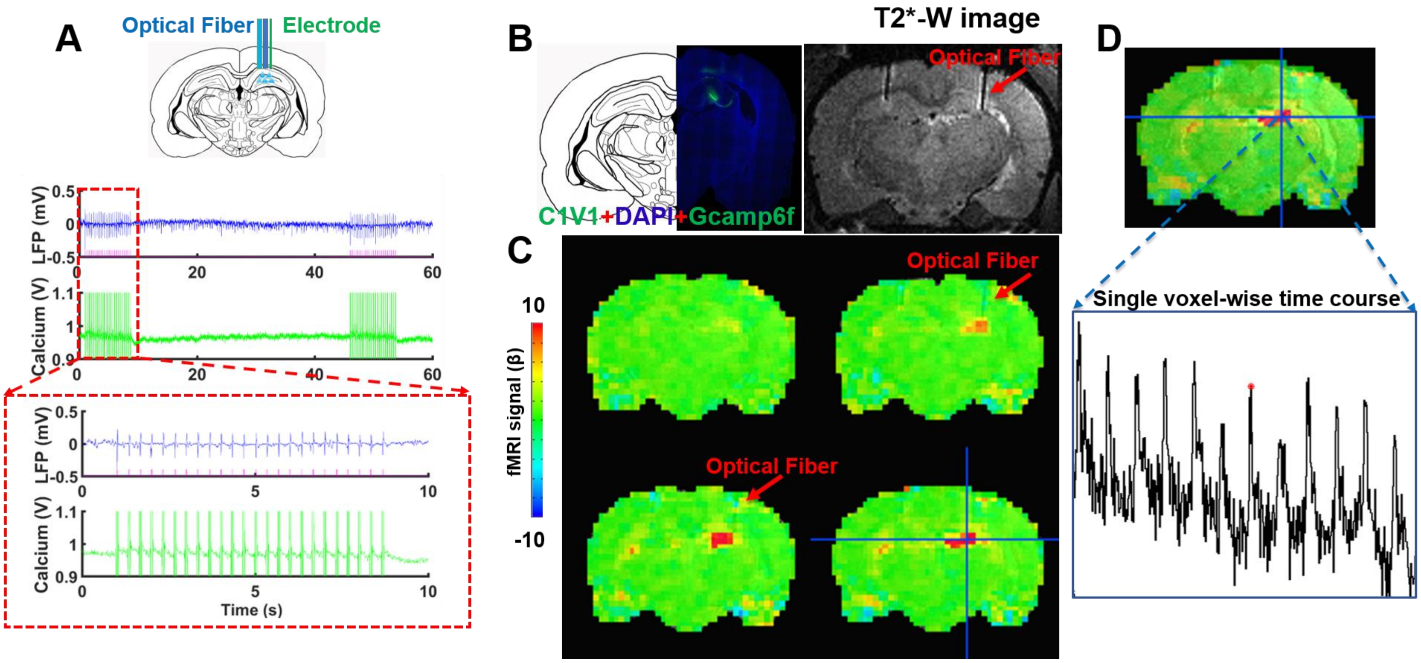

Supplementary Figure 3. Simultaneous C1V1-evoked GCamp6f-mediated Ca^{2+} recording with LFP or EPI-fMRI in the hippocampus.

A. Simultaneous LFP (blue) and Ca^{2+} signal (green) traces from neurons expressing C1V1 in the hippocampus with optogenetic stimulation (illumination: 10 ms light pulse, 3 Hz, 8 s, 5 mW, 590 nm) with enlarged view outlined in the red box.

B. Immunohistological staining of C1V1 and Gcamp6f co-expressed in the hippocampus (left), and the T2*-weighted (T2*-W) image shows the optical fiber (red arrow) inserted into the hippocampus.

C. A representative color-coded BOLD-fMRI map shows the optogenetically activated hippocampus through C1V1 (illumination: 10 ms light pulse, 3 Hz, 8 s, 5 mW, 590 nm).

D. The BOLD-fMRI time course from a single voxel in hippocampus is plotted in a block-design paradigm (12 epochs).

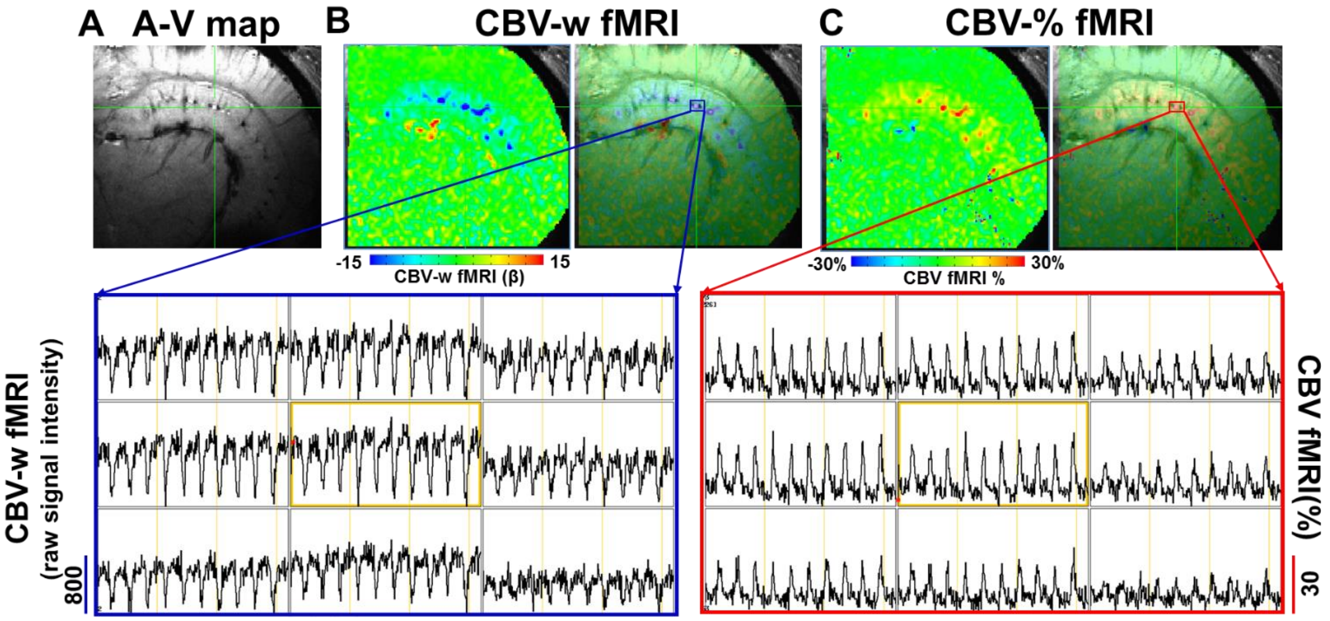

Supplementary Figure 4. The Optogenetically driven hippocampal single-vessel CBV % map.

A. Representative 2D A-V map of the hippocampal vasculature.

B. The CBV-weighted fMRI map shows the T2*-weighted signal changes from the individual hippocampal arterioles (bright dots). The time courses of the CBV-w signal from arterioles voxels (3x3 windows, blue box) show the negative signal changes per epoch of the stimulation paradigm.

C. The CBV % fMRI map shows the percent CBV signal changes from the individual hippocampal arterioles (bright dots). The time courses of the CBV % signal from arterioles voxels (3x3 windows, red box) show the positive signal changes per epoch of the stimulation paradigm.

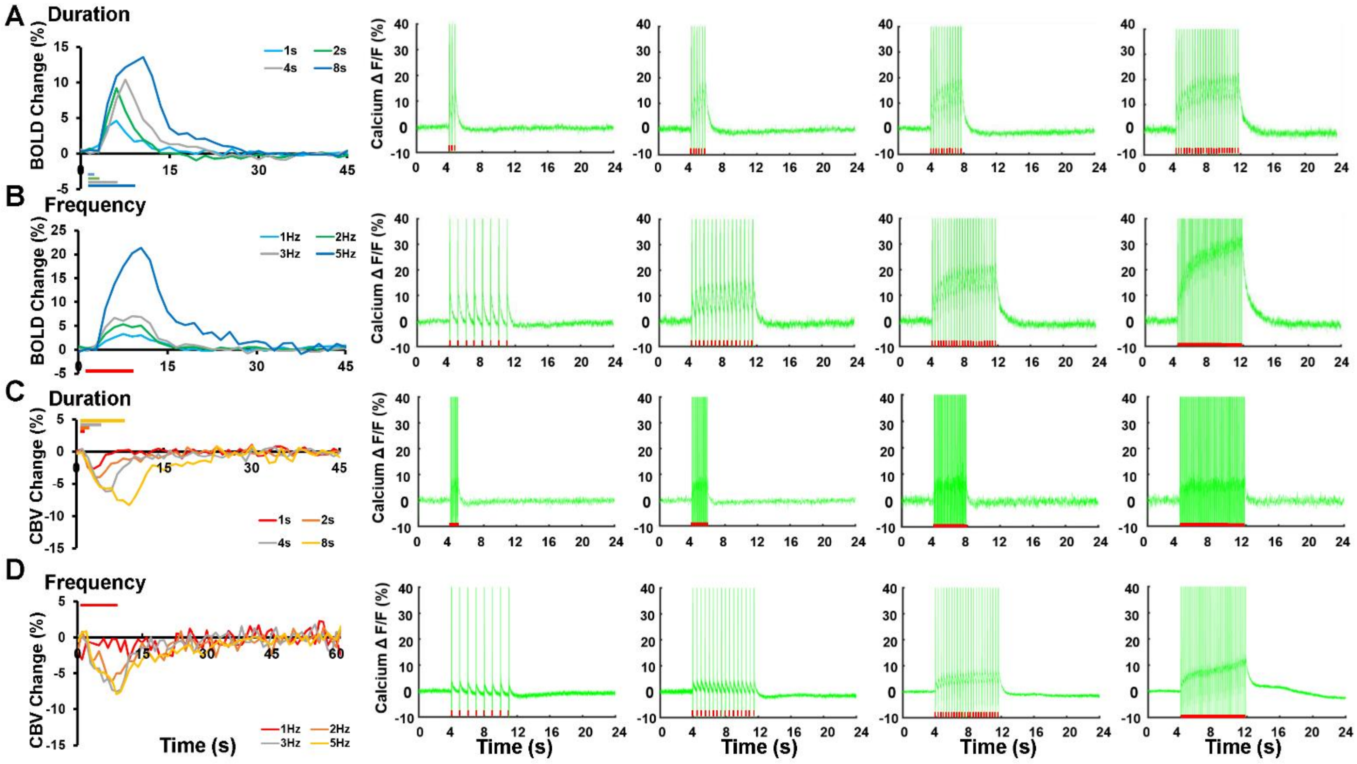

Supplementary Figure 5. Simultaneous single-vessel BOLD/CBV fMRI and Ca^{2+} recordings in the hippocampus with different optogenetic stimulation paradigms.

A. Stimulation duration-dependent BOLD-fMRI with concurrent Ca^{2+} recordings (1, 2, 4, 8 s).

B. Stimulation frequency-dependent BOLD-fMRI with concurrent Ca^{2+} recordings (1, 2, 3, 5 Hz).

C. Stimulation duration-dependent CBV-fMRI with concurrent Ca^{2+} recordings (1, 2, 4, 8 s).

D. Stimulation frequency-dependent CBV-fMRI with concurrent Ca^{2+} recordings (1, 2, 3, 5 Hz).

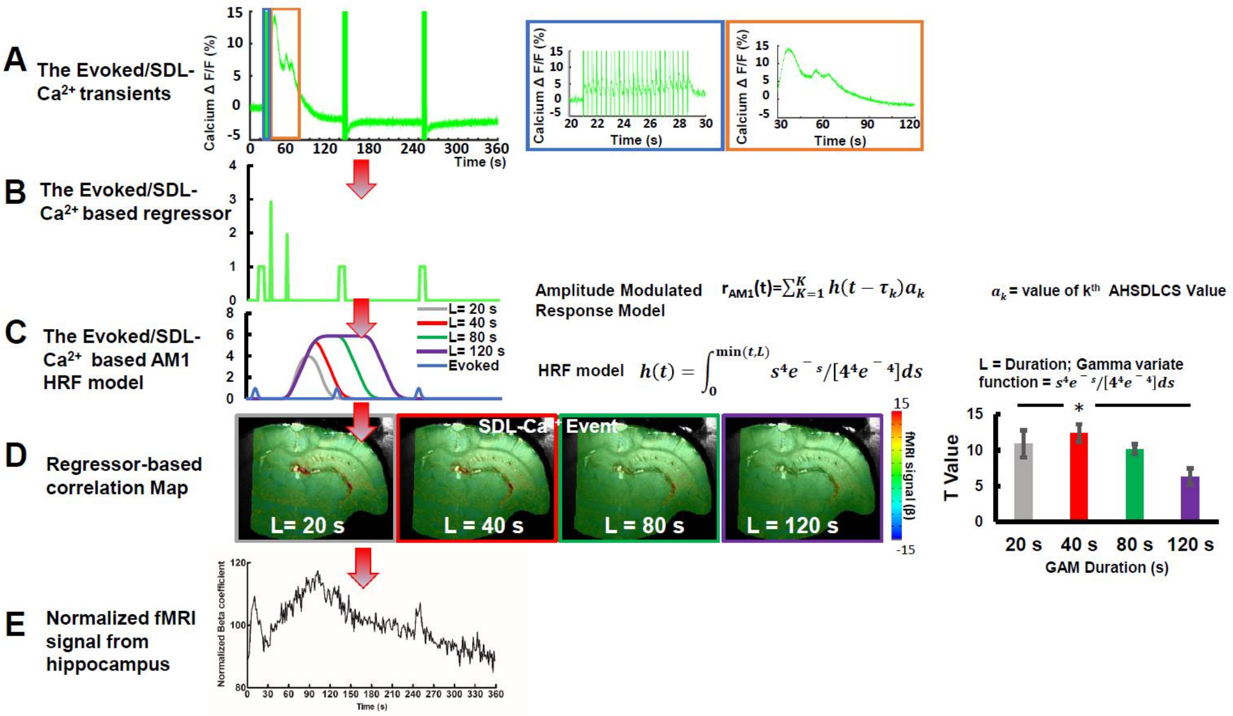

Supplementary Figure 6. The flow diagram to calculate the SDL-Ca^{2+} signal-based single-vessel BOLD fMRI correlation map.

A. Neuronal Ca^{2+} signals and single-vessel BOLD fMRI signals were acquired simultaneously during SDL-Ca^{2+} events. A representative time course of the neuronal Ca^{2+} signal shows the optogenetically evoked Ca^{2+} signal and the SDL-Ca^{2+} signal with enlarged views of these two events (blue and orange box).

B. Peak timing and amplitudes of the optogenetically evoked and SDL-Ca^{2+} events were used to create the regressors for the single-vessel BOLD fMRI correlation.

C. Amplitude modulated BOLD response models are generated base on the Evoked/SDL-Ca^{2+}-based regressors. The ideal functions (HRF models) of the representative time course of Evoked/SDL-Ca^{2+} signal are represented with varied duration (L).

D. Voxel-wise correlation maps of the single-vessel BOLD fMRI signal with the simultaneously acquired neuronal Ca^{2+} signal with the HRF models at varied duration (L), showing t statistic values at L=40 s (ANOVA, one way, F=4.93, p=0.013, n=5).

E. A representative time course of the single-vessel BOLD fMRI signal from the hippocampus shows the positive fMRI signal correlated to the occurrence of the SDL-Ca^{2+} signal.
