# Supplementary material for: Mapping optogenetically-driven single-vessel fMRI with concurrent neuronal calcium recordings in the rat hippocampus
Source: Nat Commun. 2019 Nov 20;10:5239. doi: 10.1038/s41467-019-12850-x (PMC6868210; doi:10.1038/s41467-019-12850-x)
Supplement: Supplementary file 3 — Description of Additional Supplementary Files [file 41467_2019_12850_MOESM3_ESM.docx]

**Description of Additional Supplementary Files**

File name: Supplementary Movie 1.
Description: The hippocampal vasculature and structure. The upper panel shows the saddle-like rat hippocampus in a semi-transparent 3D rendering movies with different maximal intensity projection scales. The lower movie shows the 3D MRA rendering of the hippocampal vasculature.

File name: Supplementary Movie 2.
Description: The arteriole-venule (A-V) map of the rat hippocampus. The movie shows that all the vessel voxels are bright dots at the shorter TEs, and the venules voxels become darker (blue arrows) at the longer TEs.

File name: Supplementary Movie 3.
Description: The optogenetically-driven single-vessel BOLD/CBV fMRI in the rat hippocampus. The underlay shows the A-V map as the background (dark voxels as venules, bright voxels as arterioles). The movie in the upper panel shows the optogenetically driven single-vessel BOLD- fMRI in the hippocampus. The lower movie shows the optogenetically driven single-vessel CBV-fMRI in the hippocampus.

File name: Supplementary Movie 4.
Description: Concurrent single-vessel BOLD fMRI with neuronal calcium recording (Optogenetically evoked normal vs. SDL Ca2+ spikes). The movie in the upper panel shows the single-vessel BOLD-fMRI corresponding to optogenetically evoked Ca2+ signals (3Hz, 8s on / 112s off, 3 epochs). The movie in the lower panel shows the single-vessel BOLD-fMRI corresponding to the SDL Ca2+ spikes in an on/off block design trial.
